# Stable Hg(II)-mediated base pairs with a phenanthroline-derived nucleobase surrogate in antiparallel-stranded DNA

**DOI:** 10.1007/s00775-020-01788-x

**Published:** 2020-04-11

**Authors:** Biswarup Jash, Jens Müller

**Affiliations:** Institut für Anorganische und Analytische Chemie, Westfälische Wilhelms-Universität Münster, Corrensstr. 28/30, 48149 Münster, Germany

**Keywords:** Nucleic acid, Metal-mediated base pair, Phenanthroline, Mercury

## Abstract

**Abstract:**

Metal-mediated base pairs involving artificial nucleobases have emerged as a promising means for the site-specific functionalization of nucleic acids with metal ions. In this context, a GNA-appended (GNA: glycol nucleic acid) nucleoside analogue containing the artificial nucleobase 1*H*-imidazo[4,5-*f*][1,10]phenanthroline (**P**) has already been applied successfully in a variety of homo- and heteroleptic metal-mediated base pairs, mainly involving Ag(I) ions. Herein, we report a thorough investigation of the Hg(II)-binding properties of **P** when incorporated into antiparallel-stranded DNA duplexes. The artificial nucleobase **P** is able to form Hg(II)-mediated homoleptic base pairs of the type **P**–Hg(II)–**P** with a [2 + 2] coordination environment. In addition, the heteroleptic **P**–Hg(II)–**T** pair was investigated. The addition of a stoichiometric amount of Hg(II) to a duplex comprising either a **P**:**P** pair or a **P**:**T** pair stabilizes the DNA duplex by 4.3 °C and 14.5 °C, respectively. The **P**–Hg(II)–**T** base pair, hence, represents the most stabilizing non-organometallic Hg(II)-mediated base pair reported to date. The formation of the Hg(II)-mediated base pairs was investigated by means of temperature-dependent UV spectroscopy and CD spectroscopy.

**Graphic abstract:**

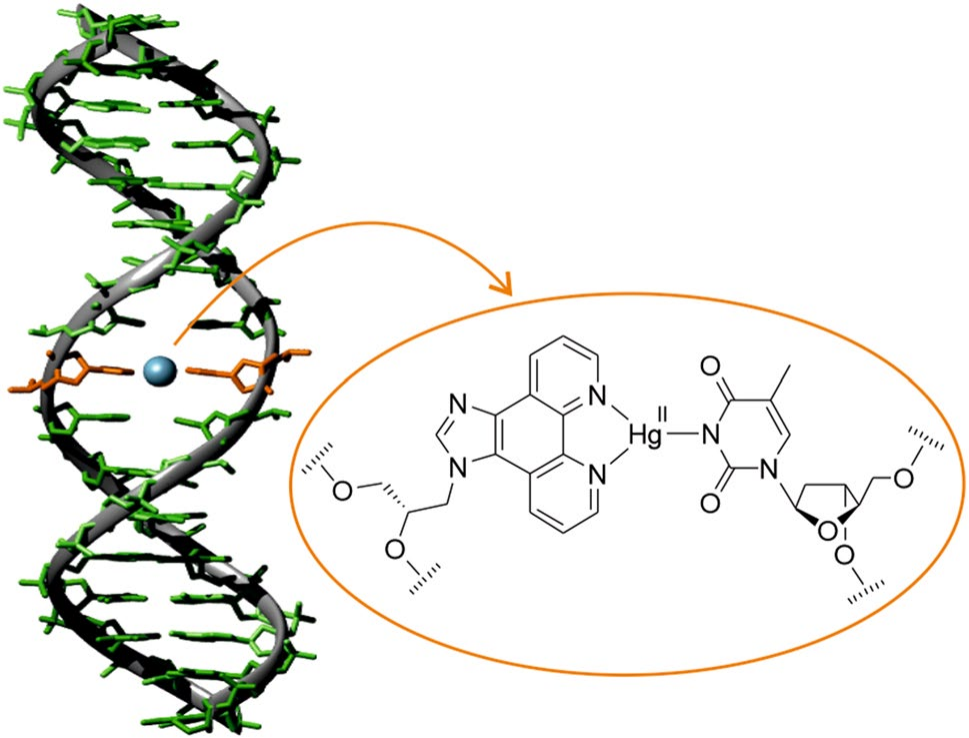

**Electronic supplementary material:**

The online version of this article (10.1007/s00775-020-01788-x) contains supplementary material, which is available to authorized users.

## Introduction

**Fig. 1:**
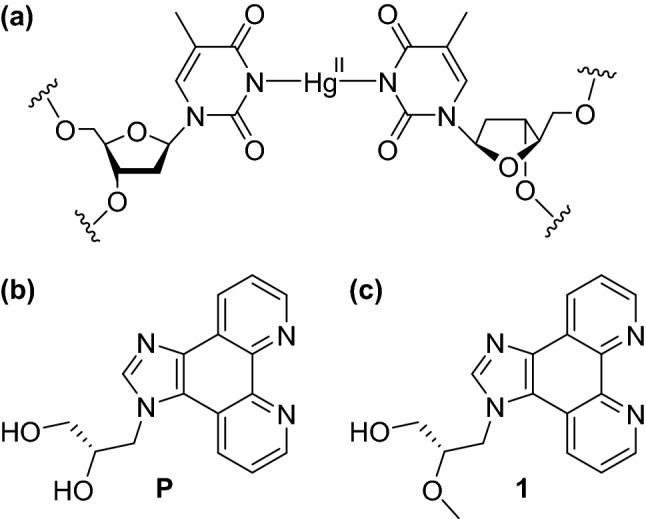
**a** Metal-mediated **T**–Hg(II)–**T** base pair, **b** artificial nucleoside analogue **P** ((*S*)-3-(1*H*-imidazo[4,5-*f*][1,10]phenanthrolin-1-yl)propane-1,2-diol), **c** model nucleobase **1** ((*S*)-3-(1*H*-imidazo[4,5-*f*][1,10]phenanthrolin-1-yl)-2-methoxypropan-1-ol)

Natural DNA is composed of four nucleobases and an anionic sugar-phosphate backbone. It forms a robust antiparallel-stranded double-helical structure based on self-assembly and molecular recognition. These unique properties render it an ideal building block in the context of supramolecular chemistry and DNA nanotechnology [1, 2]. The modular composition of DNA can be exploited to arrange metal ions within its helix in a predetermined fashion [3–5]. Historically, the interaction of transition metal ions with nucleic acids was first demonstrated using simple viscosimetry experiments of DNA solutions in the presence of various inorganic salts [6]. Subsequently, the discovery of the **T**–Hg(II)–**T** base pair pioneered the introduction of metal-based functionality into the nucleic acid scaffold (Fig. 1a) [7, 8]. The concept of applying nucleobases as ligands to locate metal ions inside a DNA duplex is nowadays referred to as metal-mediated base pairing. In such artificial base pairs, the natural hydrogen bonds between the complementary bases are formally replaced by metal–ligand coordinate bonds [9], leading to the desired site-specific functionalization. In fact, even duplexes consisting entirely of metal-mediated base pairs are feasible [10, 11]. Research on metal-mediated base pairing has not been restricted to natural nucleobases. It has rather been expanded by the introduction of artificial ligand-based nucleosides to bring in diversity in the form of site-specific functionalization [12–27], allowing the generation of different metal-induced DNA nanoarchitectures. A higher affinity of the artificial nucleosides towards a particular metal ion and the wide structural flexibility offered by them represent their largest advantages. Similarly, other nucleic acid topologies and nucleic acid analogues have been probed with respect to the site-specific incorporation of metal ions in analogy to metal-mediated base-pair formation [28–31]. Several promising applications have already been established in this area, including the construction of structures with a modified electrical response [32, 33], switchable devices [34], responsive devices [35, 36], regulated primer extension [37], the specific detection of canonical nucleobases [38, 39], the generation of DNA-templated metal nanoclusters [40], an expansion of genetic four-letter code [41], etc. Due to their accessibility from commercial resources, canonical nucleobases involved in metal-mediated base pairing have been most extensively studied, giving rise to **C**–Ag(I)–**C** and **T**–Hg(II)–**T** pairs [42]. Consequently, the first successful application of metal-mediated base pairing, namely a Hg(II)-sensor in water, was based on the well-established affinity of thymine towards Hg(II) [43]. Numerous structural studies resulted in a better understanding of the requirements for metal-mediated base-pair formation [9], indicating, for example, a structural flexibility particularly of base pairs involving monodentate ligands [44–46].

We have previously reported the use of the artificial nucleoside analogue **P** in various contexts of metal-mediated base pairing [47–50], both in antiparallel- and parallel-stranded DNA duplexes (Fig. 1b). The ligand prefers to engage in a tetrahedral coordination geometry when forming [2 + 2] coordination complexes with Ag(I) [51], Cu(I) [52], and Zn(II) [53]. On the other hand, in the presence of a complementary monodentate nucleobase, it is also compatible with a [2 + 1] coordination pattern when a *d*^10^-configured metal ion is applied [47–50]. In this report, the homo- and heteroleptic base-pair formation of the nucleoside analogue **P** in the presence of Hg(II) is presented for antiparallel-stranded DNA duplexes. The initial goal was to generate a reversibly formed Hg(II)-mediated base pair that is more stable than the conventional **T**–Hg(II)–**T** base pair, so that a nanodevice with high-performance Hg(II)-sensing capacity becomes feasible.

## Materials and methods

The (*S*)-configured imidazophenanthroline-containing GNA-based phosphoramidite and the model nucleobase **1** were synthesized according to the procedure published earlier [51]. DNA synthesis was performed in the DMT-off mode on a K&A Laborgeräte H8 DNA/RNA synthesizer. For the introduction of **P** into the oligonucleotide, an increased threefold coupling time was applied. After synthesis and purification, the desalted oligonucleotides were characterized by MALDI-TOF (matrix-assisted laser desorption-ionization time-of-flight) mass spectrometry (Table 1, Fig. S1, Supplementary material). MALDI-TOF mass spectra were recorded on Bruker Reflex IV or Bruker Autoflex Speed instruments using a 3-hydroxypicolinic acid/ammonium citrate matrix. During the quantification of the oligonucleotides, a molar extinction coefficient *ε*_260_ of 10.0 cm^2^ mmol^−1^ was used for **P**. UV measurements were performed on a CARY 100 Bio UV spectrometer using solutions containing 1 μM oligonucleotide duplex, 150 mM NaClO_4_, 2.5 mM Mg(ClO_4_)_2_, and 5 mM buffer (MOPS (pH 6.8), MES (pH 5.5) or borate (pH 9.0)). Prior to the assays, the samples were incubated with the added metal ions for 1 h at 5 °C. UV melting curves were recorded with a heating/cooling rate of 1 °C min^−1^ and a data interval of 1 °C. Absorbance was normalized according to *A*_norm_ = (*A – A*_min_)/(*A*_max_ – *A*_min_) at 260 nm. Melting temperatures were determined from a Gaussian fit of the maximum of the first derivative of the respective melting curve. The standard deviation of *T*_m_ represents the standard deviation of the Gaussian fit. CD spectra were recorded at 5 °C on a JASCO J-815 spectropolarimeter, smoothed, and a manual base line correction was applied.

**Table 1:** Oligonucleotides used for the investigation of Hg(II)-mediated base pairing

Duplex	DNA oligonucleotide sequences under investigation	Entry	[M + H]^+^/Da	
			Calcd	Found
**I**	5′-d(GAG GGA **P**AG AAA G) Chemical formula: C_136_H_158_N_64_O_68_P_12_	ODN 1	4147	4151
	3′-d(CTC CCT **P**TC TTT C) Chemical formula: C_130_H_164_N_34_O_80_P_12_	ODN 2	3853	3858
**II**	5′-d(GAG GGA **P**AG AAA G) Chemical formula: C_136_H_158_N_64_O_68_P_12_	ODN 1	4147	4151
	3′-d(CTC CCT **T**TC TTT C) Chemical formula: C_124_H_165_N_32_O_83_P_12_	ODN 3	3802	3804
**III**	5′-d(GAG GGA **A**AG AAA G) Chemical formula: C_130_H_158_N_65_O_69_P_12_	ODN 4	4105	4108
	3′-d(CTC CCT **T**TC TTT C) Chemical formula: C_124_H_165_N_32_O_83_P_12_	ODN 3	3802	3804

## Results and discussion

### Characterization of the Hg(II)-binding behaviour of P

**Fig. 2:**
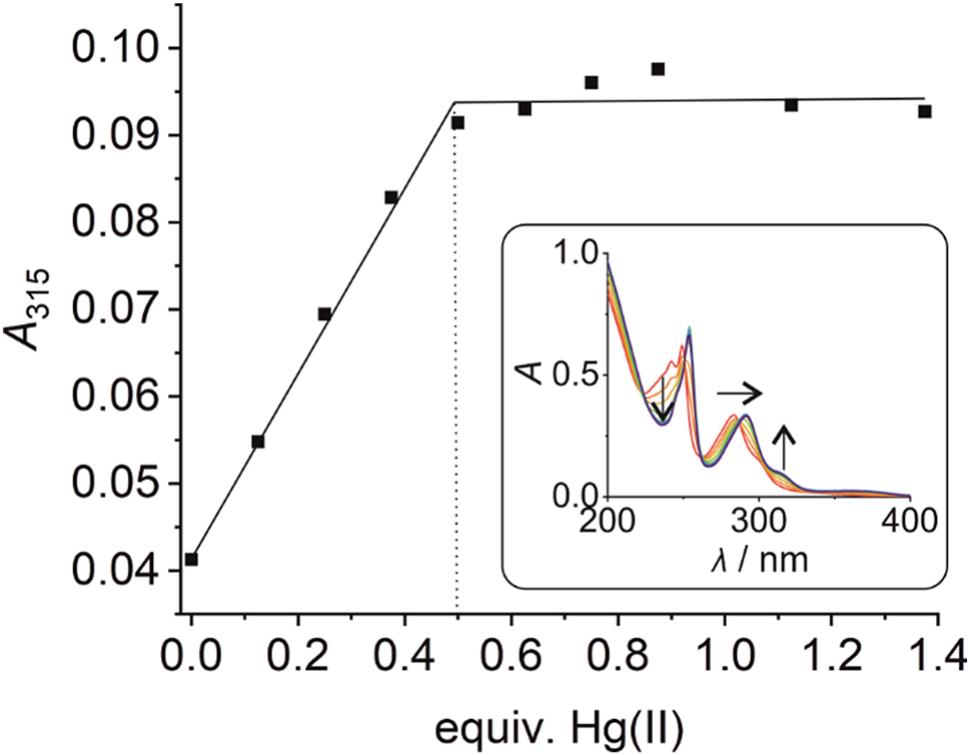
UV absorbance of an aqueous solution of compound **1** at 315 nm in the presence of increasing amounts of Hg(II) at pH 7. Inset: UV/Vis spectrum of compound **1** in the presence of various amounts of Hg(II). Arrows indicate the direction of the changes

Model nucleobases have proven to be helpful in the determination of the metal-binding behaviour of the natural nucleosides [54]. In model nucleobases, the ribose moiety of the nucleoside is formally replaced by an alkyl group, eliminating a possible interference of the hydroxyl groups with metal binding. Similarly, model nucleobases have helped to elucidate the preferred metal-binding stoichiometry of artificial nucleosides and the geometry of the metal-mediated base pair [55–58]. In the context of the Zn(II)-binding capability of **P**, a suitably *O*-protected derivative **1** had been reported previously (Fig. 1c) [53]. This model nucleobase was now also titrated with Hg(II) in aqueous medium. As can be seen in the inset of Fig. 2, the UV spectrum of **1** shows two absorption maxima at 249 nm and 283 nm prior to the addition of Hg(II) ions. Their stepwise addition causes a significant shift of the absorption maximum at 283 nm alongside a disappearance of the absorption maximum at 249 nm. The spectral changes can be attributed to the formation of a new species into the solution. Isosbestic points at 223, 251, 261, 287, and 337 nm clearly indicate a direct equilibrium between the free ligand and its Hg(II) complex. The new absorption band at ~ 315 nm is most likely due to a metal-to-ligand charge transfer, based on the availability of a low-lying π* orbital of the aromatic phenanthroline moiety. When plotting the absorbance at 315 nm against the amounts of Hg(II), a levelling-off effect at 0.5 equivalents of Hg(II) can be observed (Fig. 2), suggesting the formation of a homoleptic 1:2 metal complex in aqueous medium (pH 7).

### Investigation of the Hg(II)-binding properties of the DNA duplexes

The metal-binding ability of **P** inside antiparallel-stranded DNA duplexes was investigated in the context of both homo- and hetero-base pairs by introducing **P** as a central nucleoside surrogate into two short oligonucleotide sequences (Table 1). We selected these previously investigated sequences to allow a better comparison with other metal-mediated base pairs [47, 48], in particular because the sequence context is known to play an important role in the net stabilization of metal-mediated base pairs [49]. In this study, duplex **I** comprises one **P**:**P** homo base pair, whereas duplex **II** contains one central **P**:**T** hetero base pair. In addition, duplex **III** bearing natural base pairs only was investigated as a reference. The propensity towards the formation of a Hg(II)-mediated base pair was probed by an analysis of the thermal duplex denaturation (as derived by UV spectroscopy) and by a CD-spectroscopic analysis indicating the impact of the metal complex formation on the duplex conformation.

### UV-dependent thermal denaturation analyses

#### Homo base pair

**Fig. 3:**
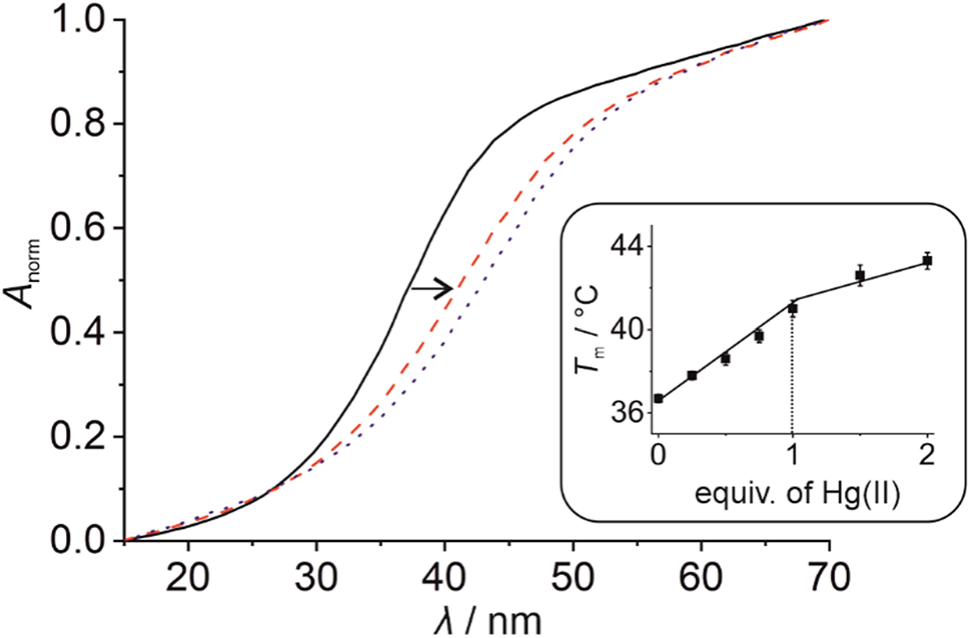
Denaturation of duplex **I** as determined UV-spectroscopically [solid black line: 0 equiv of Hg(II); broken red line: 1 equiv of Hg(II); dotted blue line: 2 equiv of Hg(II)]. The inset shows the melting temperature *T*_m_ depending on the amount of Hg(II). Experimental conditions: 1 μM duplex, 150 mM NaClO_4_, 2.5 mM Mg(ClO_4_)_2_, and 5 mM MOPS (pH 6.8)

In the absence of any Hg(II), the melting temperature of duplex **I** amounts to 36.7(2) °C (Fig. 3). For comparison, duplex **III** lacking the artificial **P**:**P** pair melts at 43.6(4) °C (Fig. S2b, Supplementary material). This difference in melting temperature *T*_m_ can most likely be attributed to the distortion around the **P**:**P** pair upon the incorporation of the bulky imidazophenanthroline moiety with an acyclic backbone into the duplex. When solutions containing duplex **I** are incubated with Hg(II) prior to the thermal denaturation analysis, a significant increase of the melting temperature *T*_m_ is observed. In the presence of one Hg(II) per duplex, a transition of the sigmoidal melting profile towards higher *T*_m_ can be observed, leading to a *T*_m_ of 41.0(4) °C. Addition of excess Hg(II) ions confers a minor additional thermal stabilization only. In combination with the established stoichiometry of the model nucleobase complex [Hg(**1**)_2_]^2+^, the incorporation of one Hg(II) into the **P**:**P** pair can be safely concluded, leading to a **P**–Hg(II)–**P** base pair. The generation of this metal-mediated base pair causes a duplex stabilization of 4.3(4) °C.

**Fig. 4:**
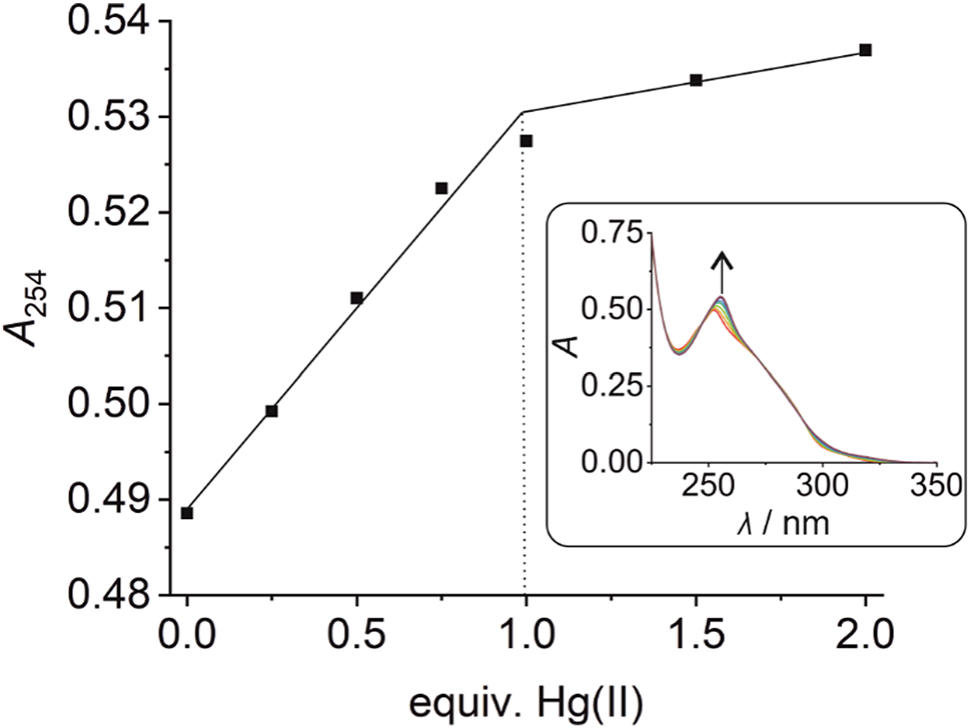
Change of the UV absorbance of duplex **I** at 254 nm upon the stepwise addition of Hg(II), clearly confirming the binding of one Hg(II) per duplex (and hence per **P**:**P** pair). Inset: UV spectrum of duplex **I** in the presence of various amounts of Hg(II). The arrow indicates the direction of the change. Experimental conditions: 1 μM duplex, 150 mM NaClO_4_, 2.5 mM Mg(ClO_4_)_2_, and 5 mM MOPS (pH 6.8)

The stoichiometry of the **P**–Hg(II)–**P** base pair within duplex **I** is further confirmed by a plot of the UV absorbance of the duplex at 254 nm vs. the added equivalents of Hg(II) (Fig. 4). Again, a drastic change in absorbance is observed up to the addition of one Hg(II) per duplex, whereas the addition of excess Hg(II) has a less pronounced effect and can most likely be attributed to non-specific binding.

#### Hetero base pair

Duplex **II** comprises essentially the same oligonucleotide sequence as duplex **I**, differing only in one nucleoside. In duplex **II**, one of the artificial nucleoside analogues **P** is formally replaced by a thymine residue. As the thymine moiety must be deprotonated at its N3 position to enable a coordination of the Hg(II) ion, the Hg(II)-binding studies were performed at different pH values.

The UV-based thermal denaturation studies of duplex **II** show a sigmoidal duplex melting with different melting temperatures depending on the pH of the medium (Fig. S3, Supplementary material). In the absence of Hg(II), the duplex becomes less stable with increasing pH. This trend had been observed previously for the same duplex under slightly different experimental conditions [i.e., in the absence of Mg(ClO_4_)_2_]. It can be explained by the fact that **P** becomes protonated under acidic conditions to form a stabilizing **P**H^+^–**T** base pair involving two hydrogen bonds (Fig. S5b, Supplementary material). Under neutral conditions, the formation of one bifurcated hydrogen bond is assumed, whereas deprotonated thymine cannot engage in hydrogen bonding with **P** at all.

**Fig. 5:**
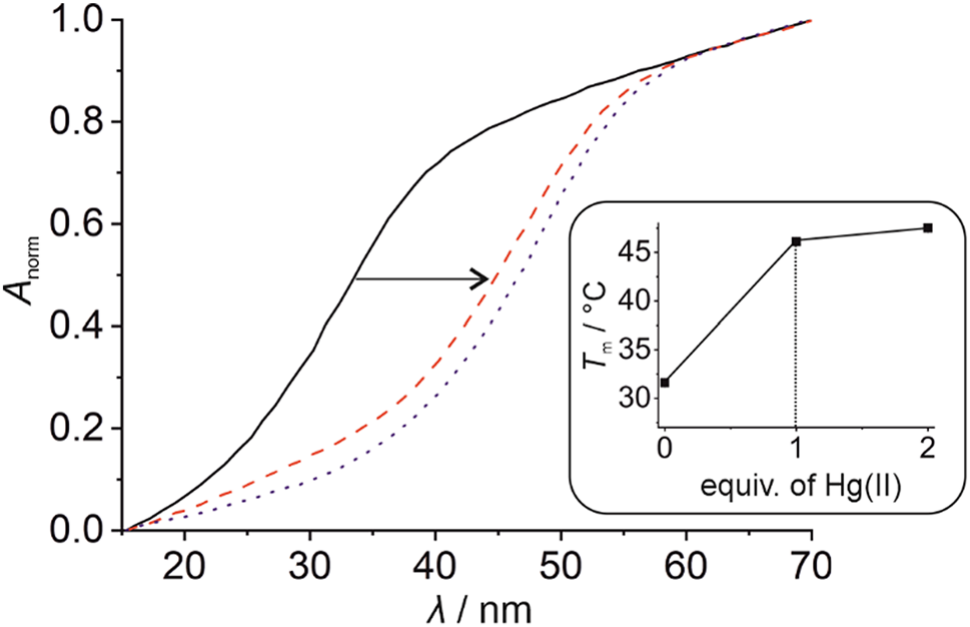
Denaturation of duplex **II** as determined UV-spectroscopically at pH 6.8 [solid black line: 0 equiv of Hg(II); broken red line: 1 equiv of Hg(II); dotted blue line: 2 equiv of Hg(II)]. The inset shows the melting temperature *T*_m_ depending on the amount of Hg(II). Experimental conditions: 1 μM duplex, 150 mM NaClO_4_, 2.5 mM Mg(ClO_4_)_2_, and 5 mM MOPS (pH 6.8)

Upon the addition of one Hg(II) per duplex, a considerable increase in the melting temperature is observed for all investigated pH values (see Fig. 5 for pH 6.8 and Fig. S3, Supplementary material, for all pH values under investigation). Again, excess Hg(II) leads to hardly any additional change in *T*_m_. These data are supportive of the formation of a mononuclear complex in the designated binding site of the duplex. To confirm the site-specific incorporation of Hg(II) into the **P**:**T** pair, additional titration experiments were performed using duplex **III**, lacking the artificial pair. Here, the addition of Hg(II) does not affect the melting temperature within the standard deviation (Fig. S2, Supplementary material), with Δ*T*_m_ essentially being 0 °C. This further corroborates the selective formation of a **P**–Hg(II)–**T** base pair in duplex **II**. Interestingly, the stability of duplex **II** bearing the **P**–Hg(II)–**T** pair strongly depends on the pH, with the *T*_m_ at pH 6.8 being significantly higher than under acidic or alkaline conditions (Table 2). The maximum increase Δ*T*_m_ of 14.5(6) °C is observed under these near-neutral conditions. Hence, the stability of duplex **II** bearing a **P**–Hg(II)–**T** pair follows the trend known for duplexes with canonical nucleobases only, in which both alkaline and acidic conditions lead to a decreased stability of the Watson–Crick base pairs. It is interesting to note that the thermal stabilization of 14.5(6) °C is more than twice as large as that observed previously for the same base pair in a parallel-stranded duplex [Δ*T*_m_ = 11 °C for a duplex comprising two **P**–Hg(II)–**T** pairs] [47]. At first, this discrepancy appears surprising, considering that fact that the base pairs should only differ in the relative orientation of their glycosidic bonds (cisoid *vs*. transoid). However, this may very well be due to the different length of the oligonucleotides under investigation, as previous publications had indicated that the thermal stabilization induced by metal-mediated base pair formation typically is larger for shorter oligonucleotides [59], as is the case here.

**Table 2:** Melting temperature *T*_m_ of duplexes **I** – **III** in the presence of various amounts of Hg(II) and change in *T*_m_ (Δ*T*_m_) upon the addition of one Hg(II) per duplex, determined at different pH values

Duplex	Base pair	pH	*T*_m_/°C 0 equiv. Hg(II)	*T*_m_/°C 1 equiv. Hg(II)	Δ*T*_m_/°C 0 → 1 equiv. Hg(II)
**I**	**P**:**P**	6.8	36.7(2)	41.0(4)	4.3(4)
**II**	**P**:**T**	5.5	32.6(4)	37.0(6)	4.4(7)
**II**	**P**:**T**	6.8	31.6(4)	46.1(4)	14.5(6)
**II**	**P**:**T**	9.0	27.6(3)	37.3(6)	9.7(7)
**III**	**A**:**T**	5.5	39.1(3)	39.1(4)	0.0(5)
**III**	**A**:**T**	6.8	43.6(4)	43.7(5)	0.1(6)
**III**	**A**:**T**	9.0	41.9(3)	40.8(7)	–1.1(8)

Previous computational studies on the mechanism of formation of a **T**–Hg(II)–**T** base pair suggested that Hg(II) acts as a Lewis acid [60]. Hence, at least one of the aqua ligands in its hydration shell becomes deprotonated. The resulting hydroxido ligand facilitates the removal of the thymine N3 proton, yielding an in situ deprotonated thymine which can then be easily mercurated. Hence, alkaline conditions are not required for the formation of a **T**–Hg(II)–**T** base pair. It is highly likely that a similar mechanism also takes place during the formation of the **P**–Hg(II)–**T** base pair in duplex **II**. Hence, an elevated pH value may not necessarily be required for the formation of the **P**–Hg(II)–**T** pair. In fact, alkaline conditions destabilize the duplex, probably due to the usual destabilization of the regular Watson–Crick pairs. The reduced stability of duplex **II** at pH 5.5 compared to pH 6.8 can likely be explained by the generally lower stability of DNA duplexes under acidic conditions, too. These explanations are in good agreement with the melting temperatures determined for duplex **III** without any artificial nucleosides.

### CD-spectroscopic analyses

CD spectroscopy was employed to elucidate the impact of the formation of the Hg(II)-mediated base pair on the secondary structure of the DNA duplexes.

#### Homo base pair

**Fig. 6:**
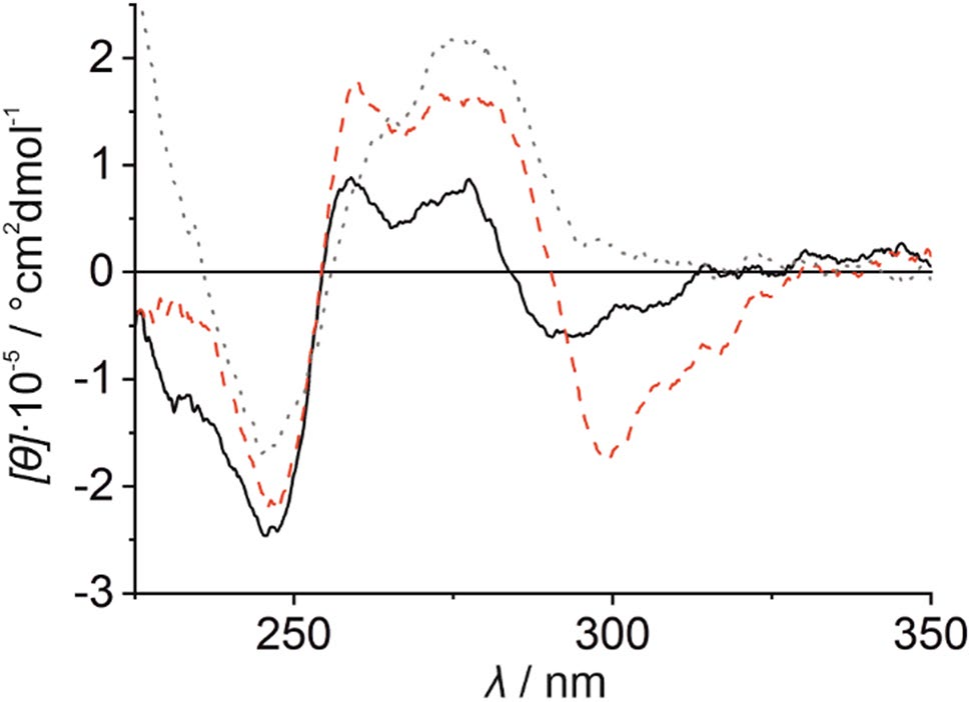
CD spectrum of duplex **I** in the absence (black solid line) and presence of one equiv of Hg(II) (red broken line). For comparison, the CD spectrum of reference duplex **III** is shown (grey dotted line), too. Experimental conditions: 1 μM duplex, 150 mM NaClO_4_, 2.5 mM Mg(ClO_4_)_2_, and 5 mM MOPS (pH 6.8)

The CD spectrum of the Hg(II)-free duplex **I** shows an intense negative Cotton effect at ca. 249 nm and two positive Cotton effects at 258 nm and 275 nm, accompanied by a broad negative Cotton effect at ca. 292 nm (Fig. 6, black spectrum). This CD spectral pattern varies significantly from that of reference duplex **III** bearing canonical base pairs only (Fig. 6, grey spectrum). The deviation from the usual B-DNA conformation must be due to the incorporation of the bulky imidazophenanthroline nucleoside analogue. Upon the addition of one equivalent of Hg(II) to the solution, the spectrum of duplex **I** (Fig. 6, red spectrum) shows a significantly more negative molar ellipticity around 300 nm along with a red shift of the characteristic Cotton effect previously found at ca. 292 nm (Δ*λ* =  + 8 nm). Such an effect had previously been seen for the Ag(I)-, Cu(I)-, and Zn(II)-mediated homo base pairs of **P** and had been assigned to the enantio-specific formation of a chiral tetrahedrally distorted metal complex [51–53]. In addition, the molar ellipticity of the two positive Cotton effects increases significantly. When performing this experiment with reference duplex **III**, no considerable changes in the CD spectra are detected (Fig. S4, Supplementary material). These observations further confirm the anticipated site-specific incorporation of the Hg(II) ion into duplex to yield a **P**–Hg(II)–**P** base pair. Due to the strong fingerprint of the chiral metal complex in the CD spectrum, a detailed conclusion regarding a possible change of the DNA duplex conformation is not possible.

#### Hetero base pair

A comparison of the CD spectra of duplex **II** under acidic, near-neutral, and alkaline conditions prior to the addition of Hg(II) shows slight differences (Fig. S5a, Supplementary material). A negative Cotton effect at ca. 295 nm is observed at pH 5.5 and 6.8, whereas no such peak is observed at pH 9.0. In contrast, a negative Cotton effect at ca. 245 nm is found under all experimental conditions, but this effect is more prominent at pH 6.8 and 9.0. It is difficult to assign these spectroscopic differences to particular changes in the DNA duplex structure. However, as previously discussed in the context of the formation of related Ag(I)-mediated base pairs [48], different hydrogen-bonding patterns between **P** and **T** are feasible, depending on the protonation state of the nucleobases and, hence, on the pH value (Fig. S5b, Supplementary material). It is, therefore, likely that **P**:**T** pairs of different structure are formed at the different pH values, giving rise to the different CD spectra of duplex **II**.

**Fig. 7:**
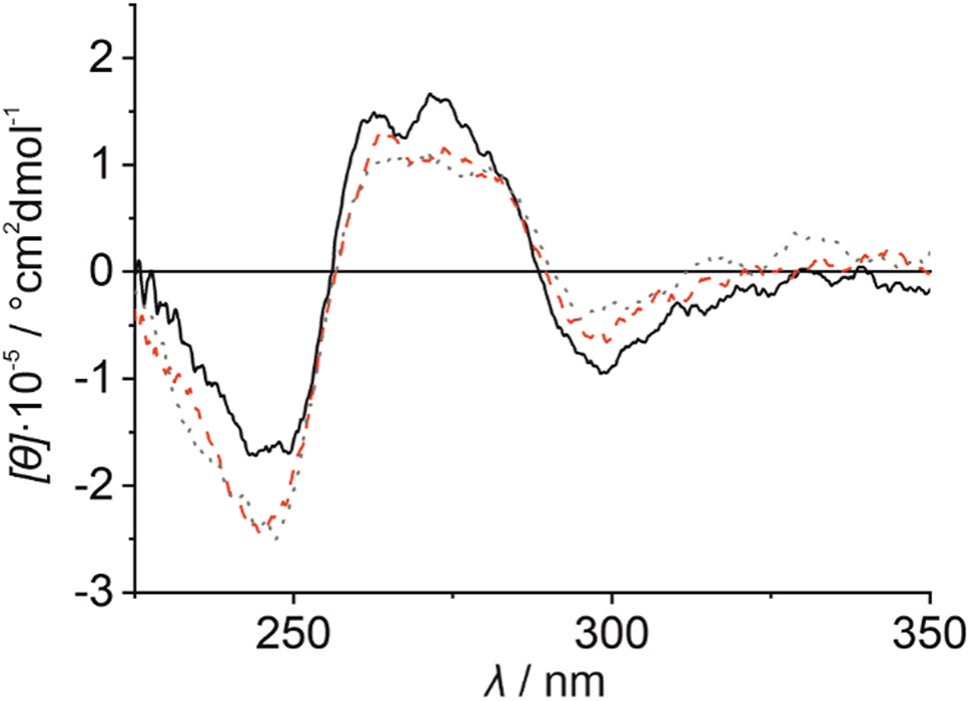
CD spectrum of duplex **II** in the presence of one equiv. of Hg(II) at pH 5.5 (solid back line), pH 6.8 (red broken line), and pH 9.0 (dotted grey line). Experimental conditions: 1 μM duplex, 150 mM NaClO_4_, 2.5 mM Mg(ClO_4_)_2_, and 5 mM buffer [MOPS (pH 6.8), MES (pH 5.5), or borate (pH 9.0)]

Interestingly, the CD spectra of duplex **II** are essentially identical in the presence of one equivalent of Hg(II) at pH 6.8 and 9.0 (Fig. 7), indicating that the same duplex conformation is adopted under these conditions upon the formation of the **P**–Hg(II)–**T** base pair. Even at pH 5.5, the same overall shape of the CD spectrum is observed, albeit with slight differences in the intensities of the Cotton effects. These differences follow the same trends as observed in the absence of Hg(II) (Fig. S5a, Supplementary material). Nonetheless, clear changes take place upon the formation of the **P**–Hg(II)–**T** base pair under all experimental conditions, as evidenced by a more negative molar ellipticity at ca. 245 nm and a less intense positive Cotton effect between 260 and 280 nm (Fig. S6, Supplementary material).

## Conclusions

The present study confirms that a **P**:**P** homo base pair within an antiparallel-stranded DNA double helix is capable of binding Hg(II), forming a **P**–Hg(II)–**P** base pair. This Hg(II)-mediated base pair significantly increases the thermal stability of the duplex. Based on the CD data, the enantio-specific formation of a chiral Hg(II) complex can be concluded. Even though the **T**–Hg(II)–**T** pair is a paradigm of a metal-mediated base pair, examples for other Hg(II)-mediated base pair are scarce. The only other instances involve pyridine [61] or organometallic-mercurated ligands [62–65]. Hence, Hg(II)-mediated base pairs formed from an artificial mispair are a rarity. The **P**–Hg(II)–**P** pair represents the first example of a Hg(II)-mediated base pair comprising a bidentate ligand and thereby increases the pool of artificial nucleobase capable of forming Hg(II)-mediated base pairs. Moreover, an antiparallel-stranded DNA duplex comprising a central **P**:**T** pair is capable of forming a highly stabilizing Hg(II)-mediated base pair. The thermal stabilization of the duplex depends on the acidity of the medium and is most pronounced at pH 6.8. Under these conditions, the **P**–Hg(II)–**T** pair turns out to be the most stabilizing non-organometallic Hg(II)-mediated base pair reported to date, with an increase in the melting temperature Δ*T*_m_ of + 14.5(6) °C. Hence, it fulfils the initial objective of creating a metal-mediated base pair that is more stabilizing than the **T**–Hg(II)–**T** base pair (Δ*T*_m_ up to 10 °C, depending on the DNA sequence) [66].

## Electronic supplementary material

Below is the link to the electronic supplementary material.

Supplementary file1 (PDF 1643 kb)
