# Supplementary material for: Stable Hg(II)-mediated base pairs with a phenanthroline-derived nucleobase surrogate in antiparallel-stranded DNA
Source: J Biol Inorg Chem. 2020 Apr 11;25(4):647–54. doi: 10.1007/s00775-020-01788-x (PMC7239801; doi:10.1007/s00775-020-01788-x)
Supplement: Supplementary file 1 — Supplementary file1 (PDF 1643 kb) [file 775_2020_1788_MOESM1_ESM.pdf]

Electronic Supplementary Information

Stable Hg(II)-mediated base pairs with a phenanthroline-derived nucleobase surrogate in antiparallel-stranded DNA

*Biswarup Jash and Jens Müller**

Westfälische Wilhelms-Universität Münster, Institut für Anorganische und Analytische Chemie, Corrensstr. 28/30, 48149 Münster, Germany. E-mail: mueller.j@uni-muenster.de

a)

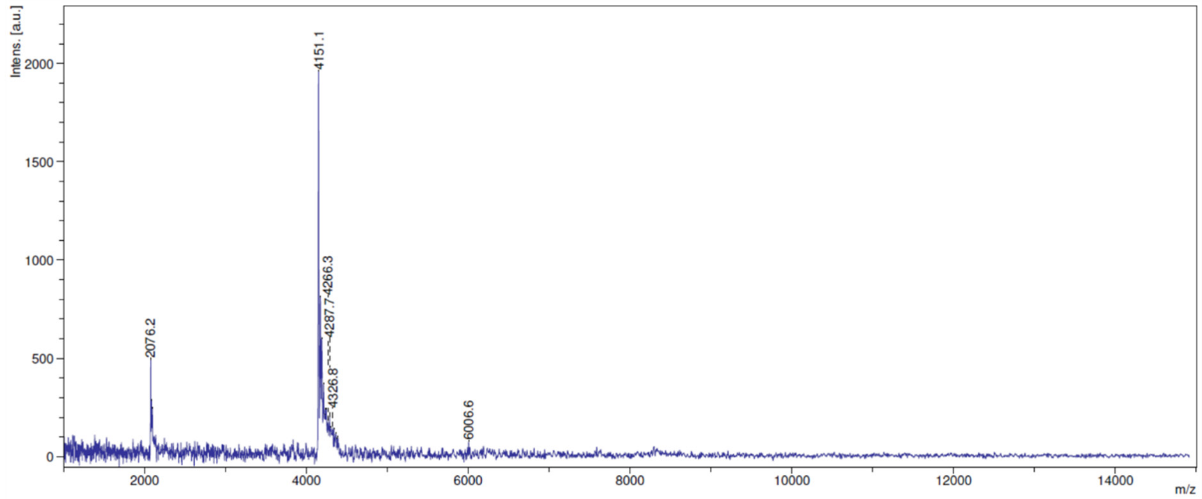
Intens. [a.u.]
2000
1500
1000
500
0
4151.1
4266.3
4287.7
4326.8
2076.2
6006.6
2000
4000
6000
8000
10000
12000
14000
m/z

b)

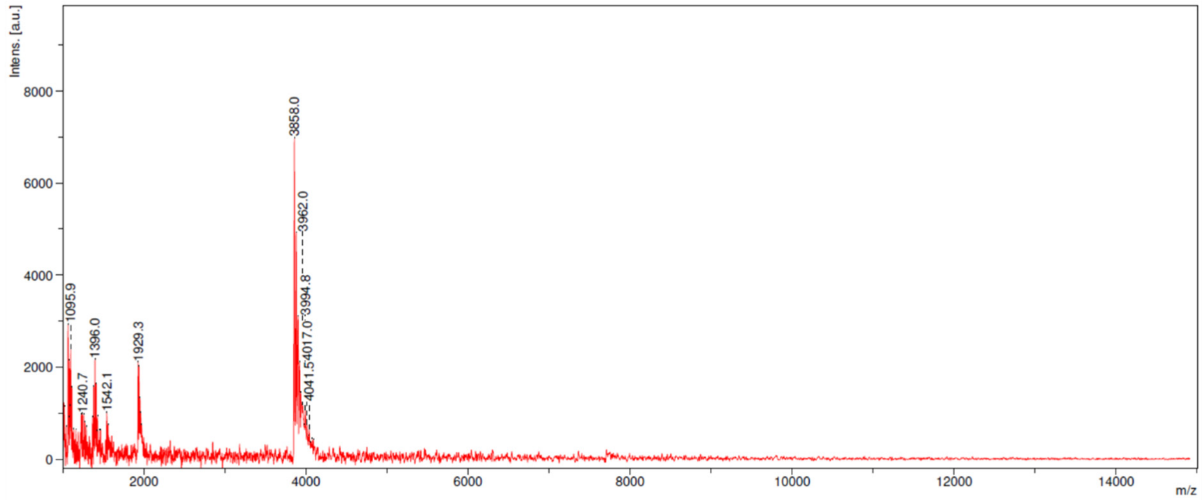
Intens. [a.u.]
8000
6000
4000
2000
0
3858.0
3962.0
3994.8
4017.0
4041.5
1095.9
1240.7
1396.0
1542.1
1929.3
2000
4000
6000
8000
10000
12000
14000
m/z

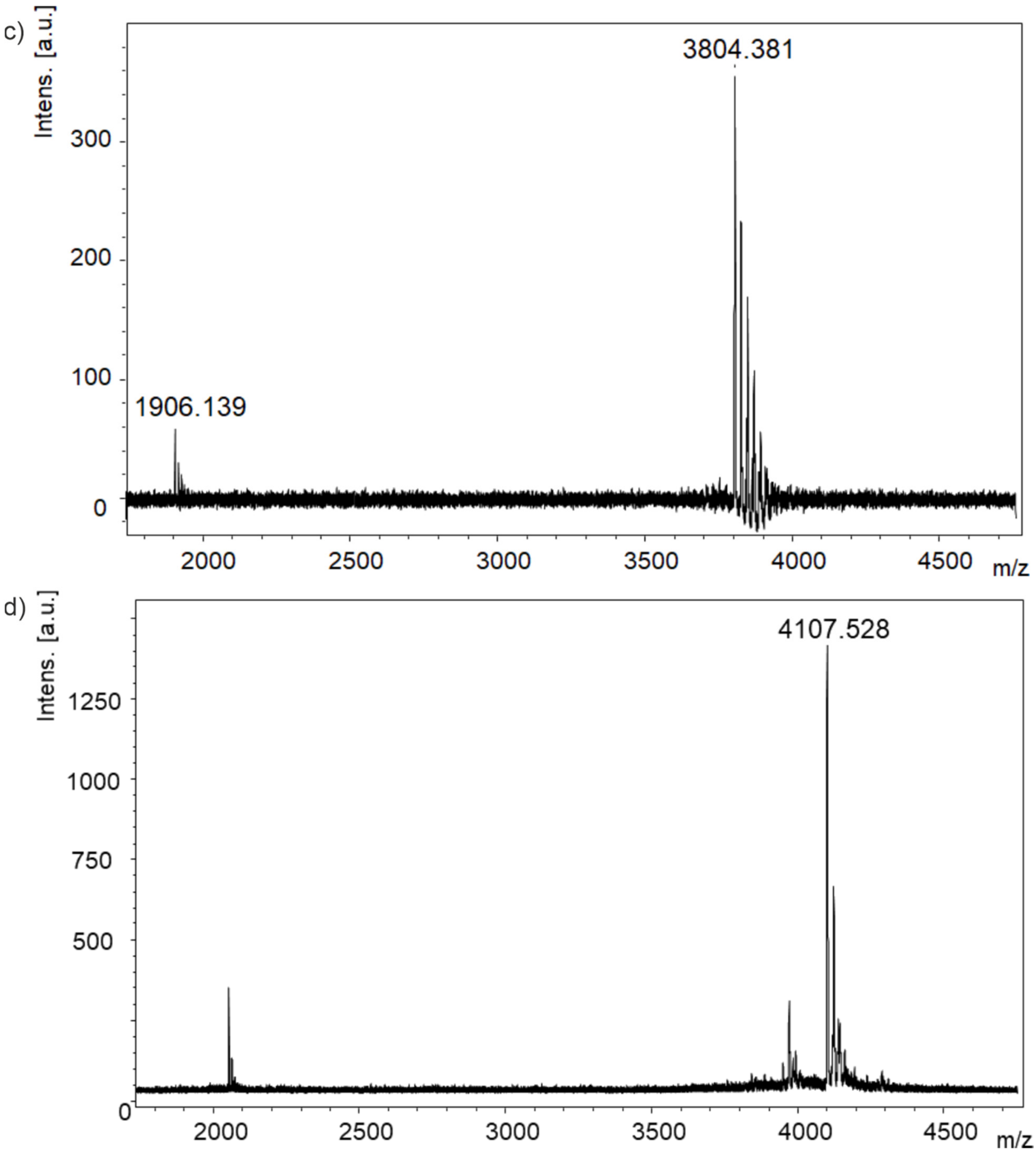

Fig. S1 MALDI-TOF spectra of oligonucleotides a) ODN1, b) ODN2, c) ODN3 and d) ODN4.

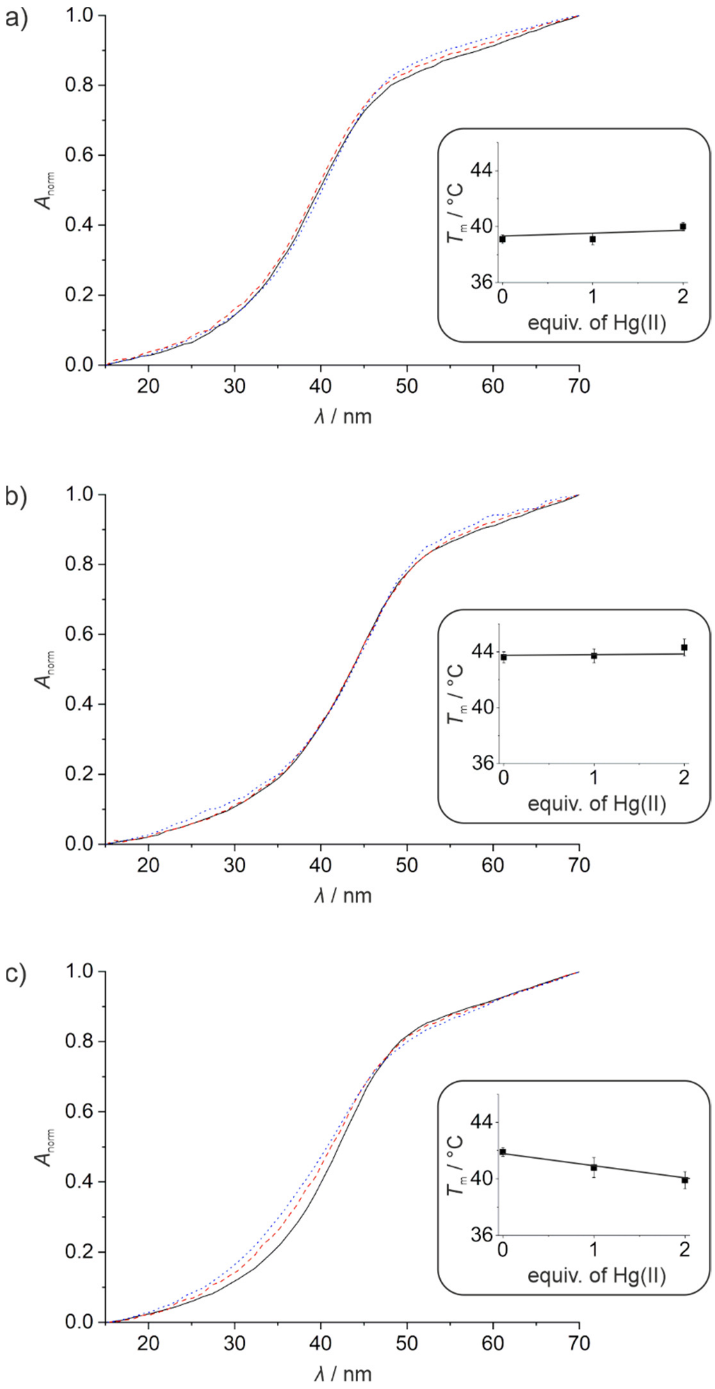

Fig. S2 Melting curves of reference duplex **III** at a) pH 5.5, b) pH 6.8 and c) pH 9.0 (solid black line: 0 equiv. of Hg(II); broken red line: 1 equiv. of Hg(II); dotted blue line: 2 equiv. of Hg(II)). The inset shows the melting temperature T_m depending on the amount of Hg(II). Experimental conditions: 1 μM duplex, 150 mM $NaClO_4$, 2.5 mM $Mg(ClO_4)_2$ and 5 mM buffer (MOPS (pH 6.8), MES (pH 5.5) or borate (pH 9.0)).

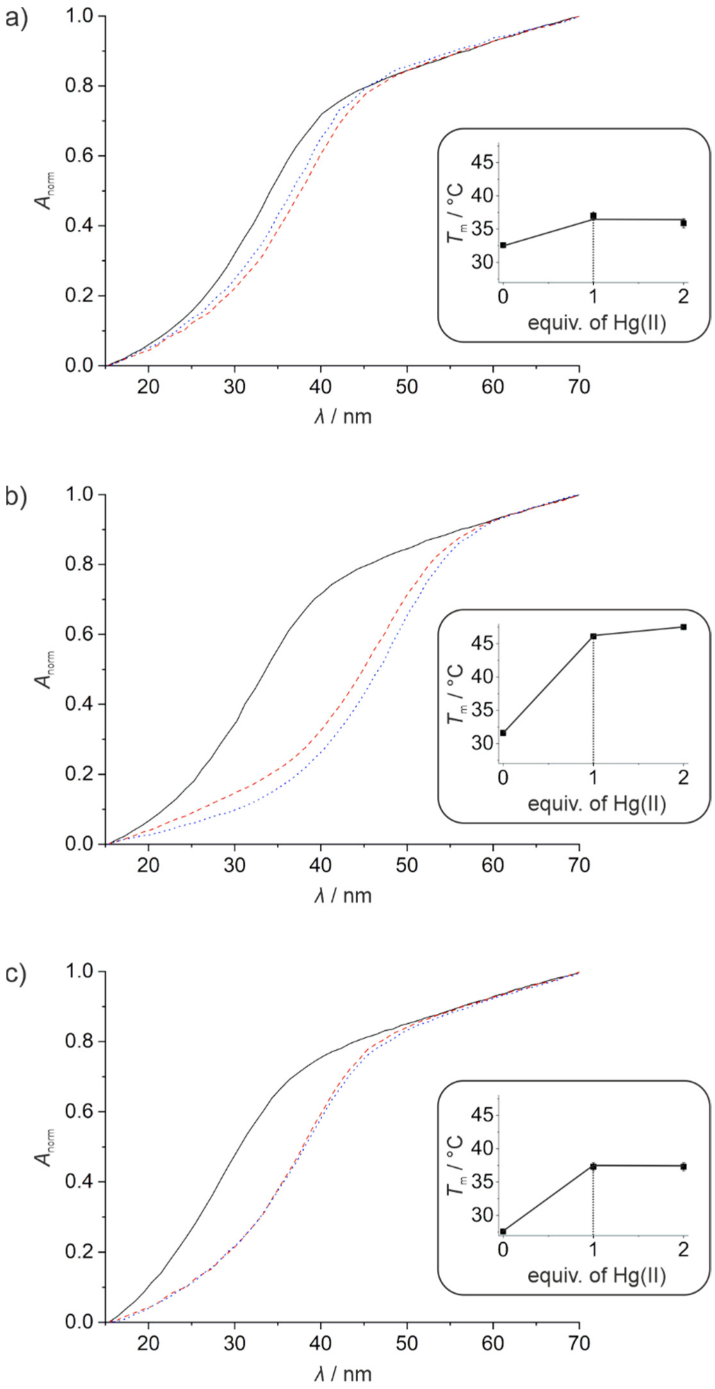

Fig. S3 Melting curves of duplex **II** at a) pH 5.5, b) pH 6.8 and c) pH 9.0 (solid black line: 0 equiv. of Hg(II); broken red line: 1 equiv. of Hg(II); dotted blue line: 2 equiv. of Hg(II))**.** The inset shows the melting temperature T_m depending on the amount of Hg(II). Experimental conditions: 1 µM duplex, 150 mM $NaClO_4$, 2.5 mM $Mg(ClO_4)_2$ and 5 mM buffer (MOPS (pH 6.8), MES (pH 5.5) or borate (pH 9.0)).

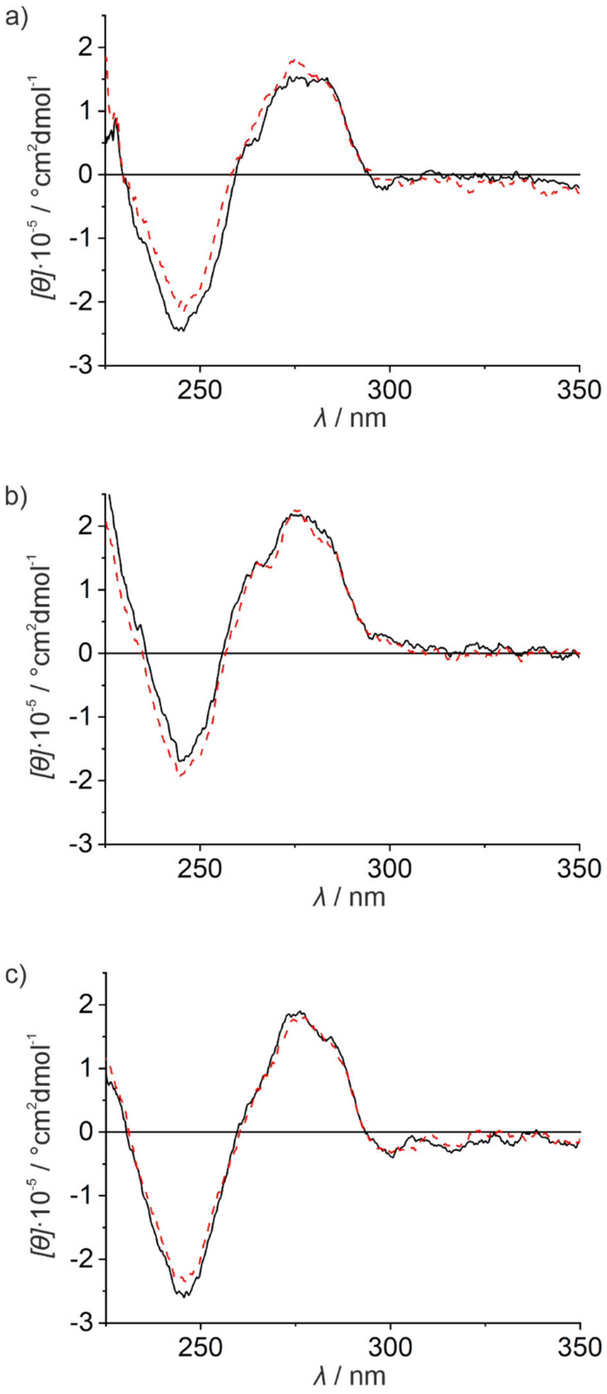

Fig. S4 CD spectra of reference duplex **III** at a) pH 5.5, b) pH 6.8 and c) pH 9.0 (solid black line: 0 equiv. of Hg(II); broken red line: 1 equiv. of Hg(II))**.** Experimental conditions: 1 μM duplex, 150 mM $NaClO_4$, 2.5 mM $Mg(ClO_4)_2$ and 5 mM buffer (MOPS (pH 6.8), MES (pH 5.5) or borate (pH 9.0)).

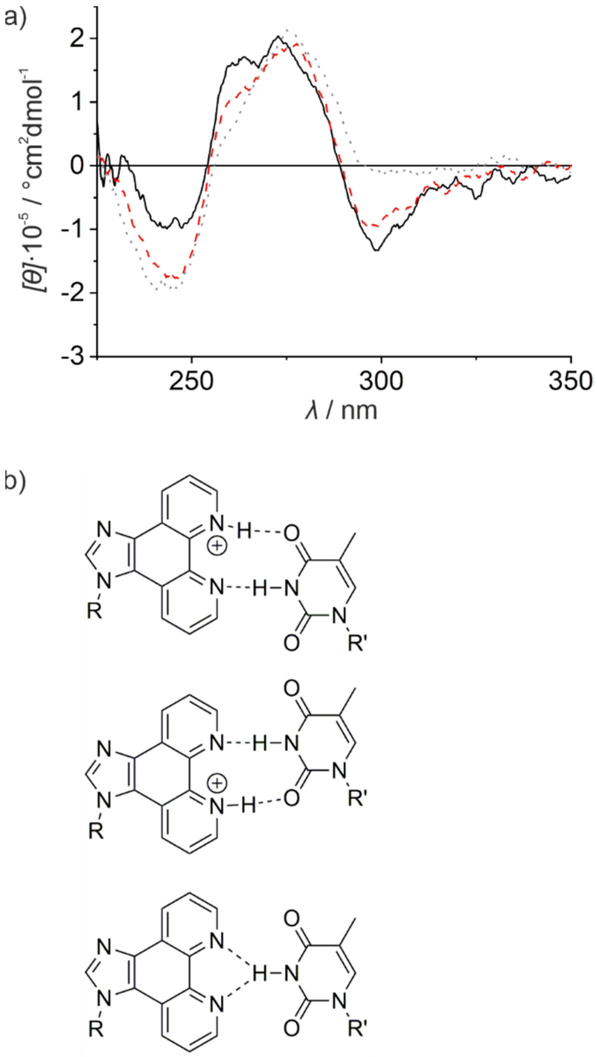

Fig. S5 a) CD spectrum of duplex **II** in the absence of Hg(II) at pH 5.5 (solid back line), pH 6.8 (red broken line) and pH 9.0 (dotted grey line). Experimental conditions: 1 μM duplex, 150 mM $NaClO_4$, 2.5 mM $Mg(ClO_4)_2$ and 5 mM buffer (MOPS (pH 6.8), MES (pH 5.5) or borate (pH 9.0)); b) Possible hydrogen-bonded base pairs involving (protonated) **P** and T.[1]

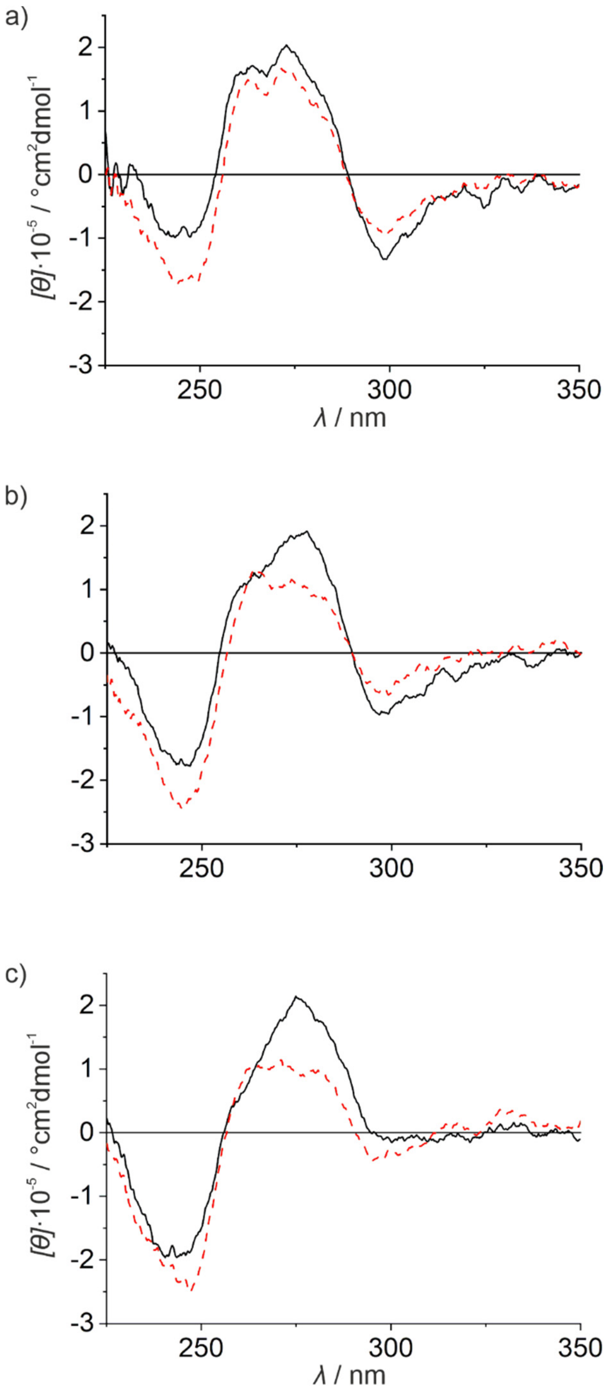

Fig. S6 CD spectra of duplex **II** at a) pH 5.5, b) pH 6.8 and c) pH 9.0 (solid black line: 0 equiv. of Hg(II); broken red line: 1 equiv. of Hg(II))**.** Experimental conditions: 1 μM duplex, 150 mM $NaClO_4$, 2.5 mM $Mg(ClO_4)_2$ and 5 mM buffer (MOPS (pH 6.8), MES (pH 5.5) or borate (pH 9.0)).

References

[1] B. Jash, P. Scharf, N. Sandmann, C. Fonseca Guerra, D. A. Megger, J. Müller, *Chem. Sci.* **2017**, *8*, 1337-1343.
